# Dimensions and Subcategories of Digital Maturity in General Practice: Qualitative Study

**DOI:** 10.2196/57786

**Published:** 2024-12-19

**Authors:** Timo Neunaber, Achim Mortsiefer, Sven Meister

**Affiliations:** 1 Health Care Informatics, Faculty of Health, School of Medicine Witten/Herdecke University Witten Germany; 2 General Practice II and Patient-Centeredness in Primary Care, Institute of General Practice and Primary Care, Faculty of Health, School of Medicine Witten/Herdecke University Witten Germany; 3 Department Healthcare Fraunhofer Institute for Software and Systems Engineering Dortmund Germany

**Keywords:** digital health, eHealth, digital maturity, maturity assessment, primary care, general practice, general practitioner, qualitative research, expert interviews, interview, explorative, dimension, subcategory, expert, care provider, content analysis

## Abstract

**Background:**

The status of the digitalization of companies and institutions is usually measured using maturity models. However, the concept of maturity in general practice is currently unclear, and herewith we examine the question of how maturity can be measured. There is a lack of empirical work on the dimensions and subcategories of digital maturity that provide information on the assessment framework.

**Objective:**

The aim of the study was to answer the question of how many and which dimensions and subcategories describe digital maturity in general practice.

**Methods:**

An explorative, qualitative research design based on semistructured expert interviews was used to investigate the dimensions of digital maturity. Twenty experts from various areas of the health care sector (care providers, interest groups, health care industry, and patient organizations) were interviewed. The interviews were analyzed based on a content-structuring analysis according to Kuckartz and Rädiker using MAXQDA software (VERBI GmbH).

**Results:**

In total, 6 dimensions with a total of 26 subcategories were identified. Of these, 4 dimensions with a total of 16 subcategories (1) digitally supported processes, (2) practice staff, (3) organizational structures and rules, and (4) technical infrastructure and were deductively linked to digital maturity. In addition to the use of digital solutions, digital maturity included, for example, individual, organizational, and technical capabilities and resources of the medical practice. The 2 further dimensions, (5) benefits and outcomes and (6) external framework conditions of the medical practice, were identified inductively with a total of 10 subcategories. Digital maturity was associated with the beneficial use of digitalization, for example, with efficiency benefits for the practice, and external framework conditions were associated with influencing factors such as the local patient situation in the medical practice.

**Conclusions:**

The results indicate that digital maturity is a multidimensional construct that is associated with many dimensions and variables. It is a holistic approach with human, organizational, and technical factors and concerns the way digitalization is used to shape patient care and processes. Furthermore, it is related to the maturity of the organizational environment as well as the benefits of a digitalized medical practice; however, this still needs to be confirmed. To measure the level of digital maturity in outpatient care as accurately as possible, maturity models should therefore be multilayered and take external influencing factors into account. Future research should statistically validate the identified dimensions. At the same time, correlations and dependencies between the measurement dimensions and their subcategories should be analyzed.

## Introduction

### Overview

“Digitalisation of health systems can significantly improve performance and outcomes” [1]. This is the conclusion reached by the Organisation for Economic Co-operation and Development in its latest Health at Glance report [2]. In its “Global Strategy on Digital Health 2020-2025,” the World Health Organization also outlines the goal of strengthening health care through the use of digital technologies [3]. Outpatient care is an essential pillar of health care systems, as it is usually the first contact patients have with health care [4]. It is not uncommon for general practitioners (GPs) to take on the role of gatekeepers who manage the patient’s subsequent care steps [5]. Due to the close networking with other service providers and institutions in the health care system, information often must be exchanged along complex care pathways. In addition to exchanging information [6], GPs can use digital technologies for a range of other purposes, such as online appointments [7] or patient treatment [8]. With the opportunities of digitalization for health care systems, calls for a measurement of digital maturity, a concept for determining the progress of digitalization, are becoming louder [2,3]. An assessment of digitalization supports policy makers in identifying gaps for investment in digital health [3]. However, this requires a deeper understanding of digital maturity, for example, for outpatient care.

### Background of Maturity Measurements

Maturity measurements are based on the use of maturity models. They are a description of a state with the aim of achieving maturity, that is, “being complete, perfect or ready” [9]. Maturity models are often based on levels that characterize the path to maturity [10,11]. They can be used for self-assessment, benchmarking, or change management, for example, by evaluating and classifying one’s own initial state based on a sequence of maturity levels [12,13]. However, this requires measurement dimensions [14]. The dimensions in turn depend on subcategories that represent second-level variables. They are determined by evaluation questions and characterize the dimensions to be measured [14]. Maturity models are used in a wide variety of areas. With the Capability Maturity Model, they were originally used for software development [15]. Application in other fields followed, such as business process management [16] or knowledge management [17]. Maturity models have also become established for measuring digital maturity in the health care sector. The Electronic Medical Record Adoption Model from the Healthcare Information and Management Systems Society is the best-known maturity model for inpatient care [18]. The World Health Organization sees potential in assessing the digital maturity of health care systems to support decisions for national funding measures [3]. In Germany, for example, the Federal Ministry of Health is promoting the measurement of the digital maturity of hospitals [19] and health authorities [20]. Despite the wide range of maturity models, their development and therefore their quality varies [14]. For example, maturity models derived from earlier models dominate [21]. Qualitative and quantitative approaches for determining the dimensions of maturity models, on the other hand, are often lacking. Therefore, empirical validation is required [22].

### Maturity Models in Outpatient Care

Maturity models and their measurement dimensions have so far been little researched for outpatient care, in contrast to inpatient care, for which Duncan et al [23], for example, have summarized measurement dimensions of maturity models. For outpatient care, there is literature about digital maturity in aged and community care [24] or commercially available options such as the Healthcare Information and Management Systems Society Continuity of Care Maturity Model [25]. For GPs or medical practices, there is currently only a diffuse understanding of digital maturity, which has only been researched in rudimentary form at best. We came to this conclusion in a recently published scoping review in which we reported an overview of the state of the research literature on the digital maturity of GPs [26]. The research primarily identified gray literature from national initiatives [27-30]. To the best of our knowledge, there are only 2 scientifically published papers dedicated to digital maturity as a whole in outpatient care and with a focus on GPs [31,32]. Teixeira et al [31] used 6 dimensions to measure digital maturity, including the use of digital systems in medical practices, as well as organizational and individual resources and abilities. The work by Haverinen et al [32] attempts to examine the level of maturity in primary and specialized care in Finland. In addition to the use of digital technologies, the regional exchange with other service providers, data security, and individual competencies of users in medical practices are also taken into account [32]. Both studies attempt to measure digital maturity but do not use validated assessment frameworks. There is no guarantee that the variables are truly measuring digital maturity. We were unable to identify any source explicitly dedicated to the validation of maturity models and their measurement dimensions for digital maturity in general practice.

### Objective

The absence of literature and the lack of empirical work on maturity models in outpatient care underscore the need for research. Although the benefits of maturity assessment for general practice as a pillar of health care systems are recognized, there is still no answer to the question of which dimensions and subcategories can be used to measure digital maturity. There is a lack of validated assessment frameworks. This is accompanied by a lack of research on dimensions of digital maturity and its subcategories. However, these are necessary to be able to make an adequate measurement. Our research question is, therefore, as follows: Which dimensions and subcategories describe the level of digital maturity in outpatient care using the example of general practice?

The overarching aim is to create the basis for further research into the level of digital maturity in outpatient care, for example, to develop validated maturity models. The specific objectives of this study are (1) to examine how many and which dimensions are associated with digital maturity in general practice and (2) to examine how many and which subcategories describe the measurement dimensions.

## Methods

### Design and Setting

An explorative qualitative research design based on semistructured, guided expert interviews was used to answer the research question. The decision to use a qualitative research design was made against the background of a lack of empirical approaches for determining the dimensions of maturity models. The aim was to examine the understanding of digital maturity independently of existing maturity models. In this respect, we opted for an inductive research approach to explicitly dedicate ourselves to researching measurement dimensions. This is intended to lay the foundation for possible later quantitative validation. The Consolidated Criteria for Reporting Qualitative Research (COREQ-32) were used to describe the results [33]. The checklist can be found in Multimedia Appendix 1.

### Participant Selection and Recruiting

The target group for the interviews was experts on digitalization in the (outpatient) health care sector in Germany. Experts are defined as people with specialized and detailed knowledge in a specific field [34]. The authors discussed whether persons meet the requirements for being experts. We selected, especially, participants who hold leading positions in the field of digitalization in health care. In our study, experts were care providers (GPs, practice managers, and medical assistants), people from interest groups (for care provider, health care industry, and health insurance companies), health care industry (eg, IT service provider), and patient organizations. We set 2 requirements for our sample. First, it should be as heterogeneous as possible in terms of age, gender, and profession. Second, most of the experts interviewed should be care providers from medical practices, as they are directly affected by digitalization. The participants were recruited in various ways. The majority were contacted by email or through LinkedIn (Microsoft). We continue to use specialist congresses and associations to recruit experts. Most recently, we were also able to recruit participants using a snowball method, whereby people who had already been interviewed recommended other experts. When selecting the experts, a parallel documentation of the sample took place, so that the authors controlled the selection of the other experts to the extent that they fulfilled requirements, for example, with regard to heterogeneity, as far as possible. In case of an expression of interest, the interview partners received a declaration of consent as well as specific information on participation. We contacted 32 people during the recruitment process. We did not receive a response from 7 people after contacting them, and we did not get a response from a further 5 people after the initial acceptance, with 1 person cancelling due to time constraints.

### Data Collection

For the expert interviews, we used an interview guide that was piloted in advance within the research team and reviewed externally by members of the target group (n=2). Based on the piloting, we refined the wording of some questions. The guide can be found in translated form in Multimedia Appendix 2. An excerpt was sent to the participants together with the information letter and the declaration of consent. The interview guide consisted of 3 sections. The first section comprised demographic questions on age, gender, occupation, and professional experience. To ensure an easy start to the interview, the second part of the guideline contained low-threshold introductory questions. These included, for example, the open question about associations with a digitalized medical practice. The third part contained specific questions on the dimensions of digital maturity in outpatient care. First, general questions were asked about possible criteria for determining digital maturity in medical practices. For the visualization and better understanding of dimensions, reference was made to the “DigitalRadar Hospital,” a current German maturity model for measuring digital maturity in inpatient care, where necessary [19]. The general question on digital maturity was further specified in the areas of “human,” “organization,” and “technology.” We derived this from Cresswell et al [35], among others, according to which digital maturity reflects organizational and human capabilities as well as technical functionalities. Although we referenced the DigitalRadar for the third part in case of comprehension problems, we deliberately decided against a stronger reference to maturity models from inpatient care in order to be able to examine the digital maturity level in outpatient care more freely. If further potentially relevant content outside of the guidelines emerged from the interview, further questions were asked. The participants were also given the opportunity to name further aspects at the end of the interview. The interviews were conducted in the period April-August 2023 exclusively by a male scientist (TN). Due to limited personal scheduling capacities of the experts, TN used Zoom (Zoom Video Communications) to conduct and record the interviews. TN also decided to use Zoom because the software met current data protection and security requirements due to its end-to-end encryption and was accepted by the participants. The software recorded both audio and video; the latter was deleted by the participant immediately after the interview was completed. Field notes were taken by TN during the interviews, for example, to record notes from participants on other experts or topics that seemed relevant. The interviews were scheduled to last from 20 to 30 minutes. On average, the interviews lasted 34 (SD 7.5) minutes. The transcription was based on the transcription rules according to Kuckartz and Rädiker [36]. Furthermore, 1 person exercised their right to comment on the transcribed interview afterward. The participant accepted the transcript on condition that a few linguistic changes were made.

### Data Analysis

The interviews were analyzed using a content-structuring qualitative content analysis according to Kuckartz and Rädiker [36]. The MAXQDA 2022 software (VERBI GmbH) was used for the evaluation [37]. The data was initially coded exclusively by the study leader. In accordance with the recommendations of Kuckartz and Rädiker [36], the main categories were first determined deductively on the basis of the topics in the interview guide. After reviewing the material, further main and subcategories were inductively expanded. To ensure the quality of the coded material, we incorporated aspects of intra- and intercoder reliability. For intracoder reliability, selected aspects of the data material were recoded after a period and compared with the original coding. For intercoder reliability, the code book was discussed together with members of the research team (n=3). All of them were members of the Chair of Health Informatics at the University of Witten/Herdecke and had several years of experience in qualitative research work. A discussion on theoretical saturation also took place in this context. We concluded that we had achieved a high level of theoretical saturation with the interviews of 20 participants. This resulted from the participants’ statements, which could be repeatedly assigned to the same main and subcategories. We therefore ruled out increasing the number of participants in view of the low added value [38].

### Reflexivity

TN, who was at the time of the study a PhD candidate, conducted the study consequently to the first study [26]. TN therefore had previous knowledge of the research gap in digital maturity in outpatient care and of maturity models in general. In this respect, there was also previous knowledge of possible dimensions of digital maturity. However, as the dimensions were researched independently of any existing maturity models, there was no influence. Instead, the previous knowledge could be used to discuss the results. For data collecting, TN had knowledge of qualitative research, which was acquired, among other things, through participation in a qualitative research workshop at the Interdisciplinary Center for Health Services Research of Witten/Herdecke University [39]. Apart from the level of awareness of people in the field of digital health in outpatient care, there were no previous relationships between the participants and TN. TN placed great emphasis on protecting the rights, privacy, and confidentiality of participants throughout the research process. This concerned, for example, statements by participants from interest groups who pointed out to the author the need for anonymity of the statements. Efforts to establish a good rapport with the participants were made throughout the study. The interviews were individually adapted to the flow of discussion made by each participant.

### Ethical Considerations

Ethics approval was obtained from the Ethics Committee of Witten/Herdecke University on March 1, 2023; no ethical or legal concerns were raised (application number S-47/2023). All participants provided complete informed consent before the interview.

## Results

### Demographics of Participants

We included a total of 20 people in our sample. Care providers such as GPs and medical assistants made up the majority (n=8), followed by people from interest groups and health care industry (n=5). In some cases, experts also took on a dual role. For example, interviewees were active in interest groups and at the same time as care providers. In these cases, we counted the main activity for the demographic data. The analysis can be seen in Table 1.

**Table 1:** Demographic characteristics of experts interviewed (N=20).

Category		Participants, n (%)
**Participant group**		
	Individual care providers (GPs^a^, practice manager, and medical assistants)	8 (40)
	Interest groups (for care providers, health care industry, and health insurance companies)	5 (25)
	Health care industry (eg, IT service provider)	5 (25)
	Patient organizations	2 (10)
**Sex^b^**		
	Male	12 (60)
	Female	7 (35)
**Age (years)**		
	30-40	10 (50)
	41-50	6 (30)
	51-60	3 (15)
	<60	1 (5)
**Total work experience (years)**		
	1-10	9 (45)
	11-20	5 (25)
	21-30	3 (15)
	<30	3 (15)

^a^GP: general practitioner.

^b^Not all respondents answered the question.

### General Results

**Figure 1:**
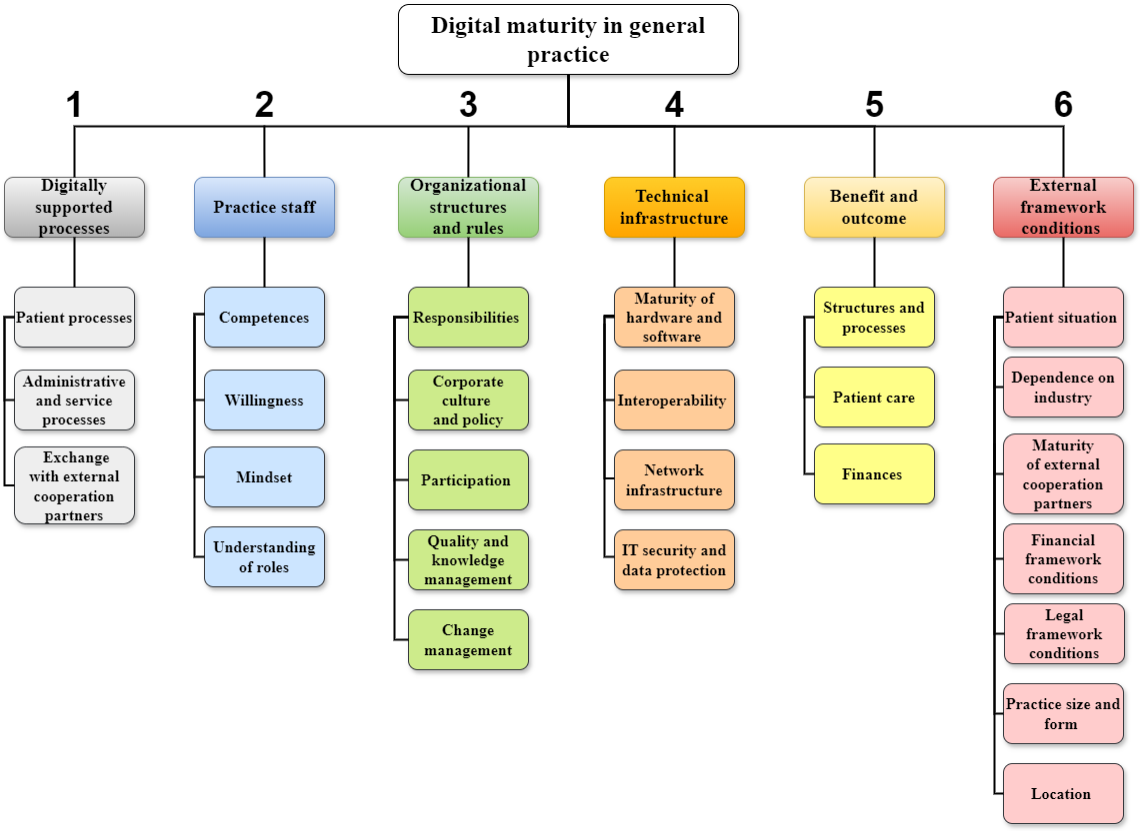
Dimensions and its subcategories of digital maturity in general practice.

The experts’ statements on possible dimensions of digital maturity could be assigned to 6 dimensions and 26 subcategories (Figure 1). Deductively, 4 dimensions could be derived that were associated with digital maturity. These included (1) digitally supported processes, (2) practice staff, (3) organizational structures and rules, and (4) the technical infrastructure of the medical practice. Two more dimensions, (5) benefits and outcome of digitalization and (6) external framework conditions of the medical practice, were identified inductively. The fewest subcategories were identified for dimension 1, digitally supported processes, and dimension 5, benefits and outcome (n=3) and the most for dimension 6, external framework conditions (n=7). The individual dimensions are examined in the following sections. Quotes from the participants are used and participant groups are assigned. A table with descriptions of every dimension and subcategory can be found in Multimedia Appendix 3.

### Digitally Supported Processes

The experts interviewed most frequently associated digital maturity with digitally supported processes (dimension 1). The experts’ statements could be assigned to 3 subcategories, each representing process areas in the medical practice. Statements on patient processes concerned the use of digital solutions in processes directly related to patients and thus essentially covered the entire patient journey from the search for a medical practice to making an appointment and treating a patient.

And then there are the classics, such as online appointment management, video consultations, in other words telemedical services that can be offered to patients, and perhaps telemonitoring services in the future, which I would perhaps also include digital health applications that patients use and through which data should also flow back to the medical practice in the future.

Participant 1, interest group

In addition to the administrative processes within a practice, the respondents also addressed communication with external organizations and made the degree of maturity dependent on the communication channel.

...i.e. the communication channels to the outside world. Are they digitalized? That would also be another dimension for me, i.e. is the exchange with other physicians, specialists, clinics, health insurance companies by post or fax or is it digitalized?

Participant 8, patient organization

### Practice Staff

The experts’ statements on digital maturity in medical practices could also be assigned to the practice staff (dimension 2). For example, digital maturity depends on the competencies of the practice staff. This included statements on the necessary knowledge of digital solutions and the ability to use them.

In my view, however, the question of whether the practice team has digital expertise is very important. If I take a medical practice with a practice owner and several medical assistants and the willingness, but also the level of knowledge about digitalization, telematics, infrastructure, IT equipment is not available, then a very big prerequisite, a very big condition for digitalization to succeed and for processes to be digitalized, is not given.

Participant 1, interest group

The individual willingness of practice staff was also identified as a factor. This is primarily related to the willingness to use digital solutions and apply them (correctly).

…but of course, there also must be a willingness among people to embrace digital processes and if there are people who block this, then the entire level of digitalization can be jeopardized or counteracted because it is simply not being lived.

Participant 7, care provider

Closely related to this were statements on the mindset of the practice staff and thus on their attitude toward digitalization.

Also, the basic attitude, of course, i.e. what do you think about digital applications, digital therapies, diagnostic applications? What do you think about the use of artificial intelligence in diagnostics? Such things could certainly also be asked.

Participant 8, patient organization

Finally, the respondents also referred to the professional role perception of the practice staff, which has an influence on the level of digitalization of a practice. Among them, 1 participant, for example, reported on physicians who did not feel obliged to deal with digitalization in accordance with their professional role.

...we simply don’t have practice managers in these medical practices, we really have physicians who are primarily there for the good of the patient and who prefer to treat patients accordingly.

Participant 19, health care industry

The understanding of professional roles was also expressed in the statement of one of the medical assistants interviewed, who on one hand was in favor of digitalization, but on the other hand would also like to maintain a personal exchange:

Of course, we now do a lot of things via the PC [personal computer], but I think it’s nice when I still know the patients and know what’s going on with them if I don’t have to look at the PC first.

Participant 18, care provider

### Organizational Structures and Rules

According to the respondents, the organizational setting with structures and rules of a medical practice also determined the level of digitalization (dimension 3). In structural terms, the organizational structure has a major influence on digital maturity. There is a need for clear responsibilities for the topic of digitalization in the team.

In our day-to-day work, we experience that everyone and no one is responsible for [digitalization] and that often causes problems because there is simply a lack of responsibility.

Participant 20, health care industry

With responsible persons, such as digitalization coordinators, change management, that is, dealing with changes in practice, could also be made more structured.

...if there is a digitalization coordinator, then the practice can respond to the new requirements of digitalization and meet them, whereas in traditional medical practices, where this is not the case, it is often an ad hoc reaction to changes, to new requirements, often with a lot of excessive demands and ignorance of the processes and procedures.

Participant 1, interest group

Last but not least, a vision for digitalization and the anchoring of digitalization in the corporate culture and policy of the medical practice were also considered important components of digital maturity.

And then there must be a commitment in practice: “Yes, we want digitalization under the maxim that we gain time.”

Participant 13, care provider

### Technical Infrastructure

When describing digital maturity, the experts interviewed referred several times to the area of technical infrastructure within a medical practice (dimension 4). The basic prerequisite for a digitalized medical practice is above all a well-developed network infrastructure.

Well, first the complete infrastructure, of course. I think without a well-developed and reliable broadband connection, it will be difficult to digitalize a medical practice, otherwise of course I won’t be able to communicate with the outside world and something like video consultations wouldn’t even be conceivable.

Participant 8, patient organization

The fulfillment of at least legal requirements in IT security and data protection is also important according to the interviewees.

..so, this degree of fulfillment of legal requirements in terms of IT security. This is also an important point because it correlates very strongly with digitalization because I would say that if a medical practice uses five systems for digitalization or for digital patient communication, for example, but is as open as a barn door, to put it bluntly, because it has no security, then in my eyes it is not a digitalized medical practice. The overall picture is part of it.

Participant 20, health care industry

Interoperable systems were also associated with a high level of maturity within medical practices. The different systems within the medical practice should be able to communicate with each other and enable data exchange.

...if examinations are carried out, also with the help of technical aids, it would of course be ideal if the sonography device, if the electrocardiogram device and the like were connected directly to the practice management system, i.e. if data is also transferred accordingly, so that there are no media breaks between the different systems.

Participant 3, interest group

However, digital maturity in medical practices was most often associated with the maturity of hardware and software in the practice and thus with factors such as usability, performance, stability, and whether the systems are up-to-date. This is primarily related to the practice management system of a medical practice.

...so, if I work with old software systems in the IT equipment, with practice management systems that are more in the category of old billing systems and not modern workflow management systems, then I would say you have a lower level of maturity.

Participant 1, interest group

Participants therefore wanted systems to be sufficiently tested before they are used in medical practices.

…it has to be applications that have been extensively tested, that simply run, to put it bluntly, that I don’t have to deal with in the practice, either as a [medical assistant] or as a physician, or that anything slows down. So, I have nothing to gain from digital processes if I have to call the service desk of my [practice management system] provider five times a day, to put it bluntly.

Participant 14, interest group

### Benefit and Outcome

In the benefits and outcomes category, the experts emphasized the meaningful and beneficial use of digitalization within the medical practice (dimension 5). A digitally mature medical practice is therefore not only characterized by the extent of digitalization but rather by the added value created by digitalization. The added value mentioned primarily related to structures and processes within the medical practice, which are associated with efficiency benefits and easier work for practice staff. One care provider interviewed emphasized this as follows:

I think we need to think about things like process optimization. Where can we really organize things in the process, in the medical practice, so that they don’t cost us so much time and so that we only see the patients we need to see? In other words, if you measure now, does the medical practice has a messenger, does the medical practice has a homepage, does the medical practice has an online appointment calendar, I say, then you can survey who uses which digital helpers, but not the degree of digitalization.

Participant 13, care provider

Closely related to this, the participants also associated a digitally mature medical practice with benefits for patient care. Digitalization must create real added value for patients.

Digitalization must not be a sure-fire success. You don’t digitalize something to digitalize it, but so that it makes work easier and relieves physicians so that they have as much time as possible for the patient. That’s what I would focus on when you examine something when you measure something. Does this save time, which in the best case is then available for the patient?

Participant 14, interest group

### External Framework Conditions

The experts interviewed mentioned external framework conditions of medical practices that could influence a possible maturity-level measurement (dimension 6). In addition to examples of dependencies on the industry or the digital maturity of external cooperation partners, the participants cited the local patient situation as an external framework condition. This should not lead to the exclusion of patients who either do not have the necessary skills or are not willing to use digital solutions. Instead, the use of digitalization must above all be adapted to the respective situation of the patient. There are situations in which a personalized approach is still necessary.

..but there is a moment when the [patient] wants to be held by another person, in the truest sense of the word, and that is the moment when I need it.

Participant 11, care provider

Another participant expressed the following concerns:

But when it comes to not being able to speak to the physician at all because you can no longer ask them any questions, then digitalization has overshot the mark again and it still must be possible.

Participant 9, patient organization

In addition to limitations due to the patient situation, respondents also referred to other local restrictions, such as the location of the medical practice.

..but it also depends on whether the medical practice is in a rural area or in a metropolitan area. This is a very relevant external factor, as is the question of the IT infrastructure in the region.

Participant 1, interest group

### Highlighted Dimensions

Although this was not explicitly part of the interview guidelines and our objective, the participants also commented on possible connections between individual dimensions and emphasized individual dimensions. Dimension 2, the role of the practice staff, was an example focus. One participant described the willingness of the practice staff as a basic prerequisite for a digitally mature medical practice.

I can have the best-equipped medical practice, but if they live well with the paper appointment calendar and don’t want to change it, they won’t change their processes, not even in the direction of their patients. Not even if the technology allows it. So, it always takes both.

Participant 1, interest group

Competences were also seen as a key factor in positive results in other measurement dimensions.

If people don’t know how to switch a computer on and off, then I probably won’t have such great results in the [...] other structures or parameters, dimensions. So, for me, the structural area, the education, the training and so on is the basis for that.

Participant 3, interest group

## Discussion

### Principal Findings

In our study, we investigated the research question of which dimensions and subcategories describe the digital maturity level in outpatient care using the example of general practice. To this end, we conducted expert interviews to understand the concept of digital maturity. Our aim was to contribute to further empirical research into maturity models in outpatient care. Our results can be interpreted as follows. First, we found that digital maturity is associated with a variety of dimensions. From the statements of the experts interviewed, we were able to derive a heterogeneous portfolio of factors ranging from the use of digital applications in processes to individual, organizational, and technical capabilities and the benefit-oriented use of digitalization. Digital transformation is therefore a multidimensional process [40]. According to Ebert and Duarte [41], digital transformation is “about adopting disruptive technologies to increase productivity, value creation, and the social welfare.” It is a departure from the original understanding of digitization, which is understood as the transformation of analog into digital processes [42]. In this respect, the results support the frequently voiced criticism of existing maturity models in inpatient care with too strong a focus on technical factors [35]. Our results indicate that the digital transformation in outpatient care is also a profound change with effects on people and organizations; practice staff and organizational structures and rules of medical practices should therefore also be explicitly considered. For example, a recent study emphasizes the influence of the personality of GPs on the digital maturity of general practices [43].

Second, a comparison between our results and those of previous studies for outpatient care allows us to derive key points for the measurement dimensions. For example, the availability and use of digital solutions in medical practices appear to be highly correlated with digital maturity. This can be seen both in our identified dimension 1 and in other maturity models or measurement approaches [31,32,44]. This result is hardly surprising, as digital processes are the result of digitalization projects in medical practices. Another focus is on the topic of capabilities, that is, what the practice staff, organization, and technical infrastructure in the medical practice can achieve [27-29,44]. For example, the competencies of physicians and medical assistants were mentioned several times in connection with digital maturity. Although previous maturity models have been criticized for focusing too heavily on technical factors, the technical infrastructure (dimension 4) nevertheless appears to play a major role in digital maturity. This is underpinned by the statements of the experts interviewed on factors such as the maturity of hardware and software or interoperability in medical practices, which can also be found in other maturity measurements [27-31].

Third, our results show similarities and differences between existing digital maturity models in other areas of health care, for example, inpatient care. While organizational, human, and technical capabilities are taken into account, management activities, such as strategy and governance, are largely missing from our results [23,45]. At most, they can be found to some extent in the area of organization in the subcategories of corporate culture and change management but were mentioned very little by the experts compared with other subcategories. In our opinion, this is because the organizational form and size of a medical practice is not comparable with that of larger organizations, such as hospitals, where management plays a greater role. In Germany, the individual practice is still the most common form of establishment in outpatient care at 58%, for which digitalization strategies are irrelevant [46]. Furthermore, digital skills and knowledge of practice management are not an integral part of the curriculum for medical students in Germany, nor are they included in the training regulations for other medical professions [47]. The job description of a practice manager is still relatively new. Last but not least, our results also underline that physicians have a major impact as role models in the digitalization of a medical practice. We conclude from the results that the digital maturity of organizational forms of outpatient care should be assessed differently than in other care areas and that management activities are still given less importance compared with actual patient care.

Fourth, our results support the sensible use of digitalization in medical practices. Although dimension 1 alone emphasizes the availability and use of digital solutions in processes, it is supplemented by dimension 5 (ie, benefits and outcomes). It is not only the frequency of use that is decisive but also the benefits for patient care or for practice staff associated with its use. This result is similar to the assessment framework of Flott et al [44] for evaluating digital maturity of health services, which was also used by Teixeira et al [31] to assess the maturity level in outpatient care. For example, the effects of digital systems on patients, structures, processes, and finances were surveyed. A decisive factor appears to be not only the frequency of use but also the associated benefits for patient care or for practice staff. However, to measure the impact of the use of digital solutions, more suitable evaluation frameworks are required. To date, there are a number of evaluation frameworks, especially for digital solutions, such as NICE (National Institute for Health and Care Excellence) in the United Kingdom, which are already attempts to bring more evidence into the digital health world [48]. With patient-reported outcome measures and patient-reported experience measures, there are more ways to measure the benefits of digital technologies that explicitly take the patient’s perspective into account [49]. It remains to be seen whether assessment approaches for the benefits of individual digital solutions are suitable for assessing the impact of digitalization in medical practices. Assessment approaches that deal more comprehensively with the circumstances of the organizational form exist, for example, the workflow composite score for hospitals [50]. With reference to information logistics, hospitals are examined according to the extent to which their IT systems describe the availability of data and information at the point of care. To the best of our knowledge, there are no explicit approaches for assessing digitalization in medical practices. If these are available, in our opinion the question remains open as to whether the effects of digitalization should be considered as causes or consequences of the digital maturity level of medical practices. In this respect, follow-up studies on the correlations between digital maturity and effects need to be carried out.

Fifth, our identified dimension 6 indicates that digital maturity is highly dependent on local circumstances such as the patient situation and other external factors that cannot be directly influenced. This poses a challenge for maturity models, as they allow little flexibility due to predefined maturity levels and a predefined maturity target. However, the statements of the experts interviewed confirm that the requirements for digital maturity in medical practices can vary. They therefore support calls for a differentiated maturity level measurement that takes local circumstances into account [35,51]. Cresswell et al [35], for example, are in favor of maturity models in which the institutions themselves define the goal of digital maturity and align it with local needs. Otherwise, maturity measurements would lead to frustration if prescribed targets are unrealistic and therefore cannot be achieved [35]. Furthermore, it can be concluded from the results for dimension 6 that dimensions and subcategories of the digital maturity of medical practices are dependent on the maturity of other players in the health care system. An example is the exchange with service providers and institutions, such as public authorities, whose digital maturity for networking is often not fully developed. A joint digitalization of institutions in the health care sector is therefore also required. This is the only way to achieve integrated care in the health care sector [35,44,51]. It is therefore also necessary to assess the digital maturity of other institutions, which in turn must consider networking with institutions such as medical practices. Other examples of the dependence of digital maturity on external framework conditions are digital health literacy and the care needs of patients. The participants interviewed expressed that the pursuit of digital maturity should not lead to patients being excluded from health care. These are important statements for the use of maturity models, as there is a risk of neglecting disadvantaged population groups with increasing digitalization [52-54]. However, high-quality health care also means equitable care that is accessible to everyone [55]. The findings on the various external framework conditions support the diverse effects of digital transformation on society. Digital maturity is therefore not just a concern of individual care areas such as general practice but must be considered in the context of a task for society.

Finally, our brief digression makes it clear that there can also be more significant dimensions within the many dimensions and subcategories of digital maturity. For example, a positive change in 1 dimension could also have an impact on other factors. The results also point to a possible weighting of factors within the construct of digital maturity. They motivate further research to identify the dimensions that have the greatest impact on digital health when promoted by national institutions.

### Future Implications

Our study builds on previous research and can in turn be used for a range of other research and applications. First, future quantitative studies should statistically test our identified dimensions. Our study did not aim to investigate statistical representativeness. However, it is necessary to contribute to a validation of the dimensions. In the context of quantitative research, correlations between individual dimensions should also be investigated in more detail. Second, future research should investigate the measurement dimensions we have identified individually. The depth of content for the individual dimensions was limited within the scope of this study. It may therefore make sense to investigate individual dimensions in more detail in studies to gain further insights into the measurement of digital maturity. Third, further studies should be carried out to examine the transferability of the dimensions to outpatient care. Digital maturity should also be analyzed for other care providers such as specialists. Finally, our results can be used in conjunction with quantitative studies for the construction of maturity models. Maturity models can be used by decision makers in the health care sector to promote digitalization in outpatient care.

### Limitations

The decision in favor of a qualitative research design enabled the largely unbiased investigation of dimensions detached from any reference models of digital maturity. Nevertheless, our conclusions must be seen in the context of the limitations of our chosen research design. In principle, the results of qualitative research are difficult to verify [56]. Control over the content essentially lies with the participants. The statements made are also subjective and are based on the personal experience and knowledge of the interviewees. Due to participants with dual professional roles, limitations in the allocation to groups of people should also be mentioned. Interpretations are therefore only possible to a limited extent. We tried to meet the challenges by deciding in favor of piloted guided interviews. In this way, we wanted to give the statements of the experts interviewed more structure. However, in favor of more structure, we cannot rule out the possibility that we have somewhat restricted the scope for freer answers as a result. Nevertheless, we were able to inductively derive 2 further dimensions despite the guidelines. Furthermore, we opted for a heterogeneous sample with actors from different areas of the health care sector. In this way, we wanted to counteract the dependence on activity-related interest formation. Our results support the literature, according to which digital transformation is a multidimensional process, so that we feel that our approach of interviewing different professional groups has been confirmed. Limitations can also be seen in our approach to data collection. Although we discussed the coding book with the members of the research team, there may be limitations in the reliability of the coding. In addition, the interview participants were all from Germany. This means that no international maturity models could have been considered by the experts. However, the aim of the study was to examine the level of digital maturity in outpatient care largely independently of existing maturity models. Instead, the understanding of digital maturity was to be explained by the experts on the basis of their knowledge and experience, for example from everyday clinical practice. This study does not claim to have researched validated measurement dimensions. This requires objective results, which are achieved by means of quantitative, statistical analyses. However, mathematical analyses were not the aim of this study and can build on our research findings. Finally, we deliberately chose to focus on general practice in this study, as it plays a key role in outpatient care. An extension to all service providers in outpatient care would have gone beyond the scope of this study. The results of our study should therefore be examined for an extension to all outpatient care.

### Conclusions

This study provides empirically analyzed dimensions and subcategories for measuring the level of digital maturity in outpatient care using the example of general practice. It is the first scientific study explicitly dedicated to the digital maturity level of general practice in a qualitative research design. The results indicate that digital maturity is a multidimensional construct and is related to human, organizational, and technical factors. Furthermore, they show that digital maturity influences the entire medical practice and thus the way digitalization is used to shape patient care. Digital maturity is also associated with external conditions of the medical practice as well as benefits of a digitalized medical practice, which, however, still need to be confirmed. In order to measure the level of digital maturity in outpatient care in medical practices as accurately as possible, maturity models should therefore be multilayered and also take external influencing factors into account.

## Appendix

Multimedia Appendix 1 COREQ (Consolidated Criteria for Reporting Qualitative Research) checklist.

Multimedia Appendix 2 Translated semistructured interview guide.

Multimedia Appendix 3 Descriptions of dimensions and subcategories of digital maturity in general practice.

## Abbreviations

COREQ-32: Consolidated Criteria for Reporting Qualitative Research
GP: general practitioners
NICE: National Institute for Health and Care Excellence
