# Supplementary material for: Dimensions and Subcategories of Digital Maturity in General Practice: Qualitative Study
Source: J Med Internet Res. 2024 Dec 19;26:e57786. doi: 10.2196/57786 (PMC11695950; doi:10.2196/57786)
Supplement: Multimedia Appendix 1 [file jmir_v26i1e57786_app1.docx]

**Multimedia Appendix 1: Completed Consolidated Criteria for Reporting Qualitative Research (COREQ) checklist.**

| **DOMAIN** | **ITEM** | **GUIDE QUESTIONS / DESCRIPTIONS** | **REPORTED IN SECTION** |
| --- | --- | --- | --- |
| **DOMAIN 1: RESEARCH TEAM AND REFLEXIVITY** | | | |
| ***Personal characteristics*** | | | |
| Interviewer/facilitator | 1 | Which author/s conducted the interview or focus group? | see “Authors Contributions” |
| Credentials | 2 | What were the researcher's credentials? (e.g., PhD, MD) | TN holds a Master of Arts in Health Care Management. |
| Occupation | 3 | What was their occupation at the time of the study? | TN is a PhD candidate. |
| Gender | 4 | Was the researcher male or female? | TN is male. |
| Experience and training | 5 | What experience or training did the researcher have? | TN was experienced in conducting interviews based on his educational background in Health Care Management. He also participated in qualitative research workshops at the Interdisciplinary Center for Health Services Research of Witten/Herdecke University. |
| ***Relationship with participants*** | | | |
| Relationship established | 6 | Was a relationship established prior to study commencement? | see “Reflexivity” |
| Participant knowledge of the interviewer | 7 | What did the participants know about the researcher? (e.g., personal goals, reasons for doing the research) | see “Reflexivity” |
| Interviewer characteristics | 8 | What characteristics were reported about the interviewer/facilitator? (e.g., bias, assumptions, reasons, and interests in the research topic) | see “Reflexivity” |
| **DOMAIN 2: STUDY DESIGN** | | | |
| ***Theoretical framework*** | | | |
| Methodological orientation and theory | 9 | What methodological orientation was stated to underpin the study? (e.g., grounded theory, discourse analysis, ethnography, phenomenology, content analysis) | see especially “Design and Setting” and “Data Analysis” |
| ***Participant selection*** | | | |
| Sampling | 10 | How were participants selected? (e.g., purposive, convenience, consecutive, snowball) | see “Participant selection and recruiting” |
| Method of approach | 11 | How were participants approached? (e.g., face-to-face, telephone, mail, email) | see “Participant selection and recruiting” |
| Sample size | 12 | How many participants were in the study? | see “Participant selection and recruiting” |
| Non-participation | 13 | How many people refused to participate or dropped out? Reasons? | see “Participant selection and recruiting” |
| ***Setting*** | | | |
| Setting of data collection | 14 | Where was the data collected? (e.g., home, clinic, workplace) | see “Data Collection” |
| Presence of non-participants | 15 | Was anyone else present besides the participants and researchers? | see “Data Collection” |
| Description of sample | 16 | What are important characteristics of the sample? (e.g., demographic data, date) | see “Participant selection and recruiting” and see “Table 1”. |
| ***Data collection*** | | | |
| Interview guide | 17 | Were questions, prompts, guides provided by the authors? Was it pilot tested? | see “Data Collection” |
| Repeat interviews | 18 | Were repeat interviews carried out? If yes, how many? | No repeat interviews were carried out. |
| Audio/visual recording | 19 | Did the research use audio or visual recording to collect the data? | see “Data Collection” |
| Field notes | 20 | Were field notes made during and/or after the interview or focus group? | see “Data Collection” |
| Duration | 21 | What was the duration of the interviews or focus group? | see “Data Collection” |
| Data saturation | 22 | Was data saturation discussed? | see “Data Analysis” |
| Transcripts returned | 23 | Were transcripts returned to participants for comment and/or correction? | see “Data Collection” |
| **DOMAIN 3: ANALYSIS AND FINDINGS** | | | |
| ***Data analysis*** | | | |
| Number of data coders | 24 | How many data coders coded the data? | see “Data Analysis” |
| Description of the coding tree | 25 | Did authors provide a description of the coding tree? | see “Multimedia Appendix 3” |
| Derivation of themes | 26 | Were themes identified in advance or derived from the data? | see “General results” |
| Software | 27 | What software, if applicable, was used to manage the data? | see “Data Collection” |
| Participant checking | 28 | Did participants provide feedback on the findings? | see “Data Collection”. We didn’t ask for feedback on the findings from participants. Nevertheless, more than half of the participants stated that they would like to learn more about the results of the study. |
| ***Reporting*** | | | |
| Quotations presented | 29 | Were participant quotations presented to illustrate the themes/findings? Was each quotation identified? (e.g., participant number) | see “Results”. Quotes from the participants were reproduced to illustrate the topics. Each quote was assigned to the number of the interview and the user group. |
| Data and findings consistent | 30 | Was there consistency between the data presented and the findings? | see “Results”. Data presented and findings are consistent. The quotes explicate the themes as presented. |
| Clarity of major themes | 31 | Were major themes clearly presented in the findings? | see “Results”. The section provides an overview of identified dimensions and sub-categories of digital maturity. We used representative quotes. |
| Clarity of minor themes | 32 | Is there a description of diverse cases or discussion of minor themes? | see “Results”. The section provides an overview of identified dimensions and sub-categories of digital maturity. We described different cases and used representative quotes. |
