# Supplementary material for: Dimensions and Subcategories of Digital Maturity in General Practice: Qualitative Study
Source: J Med Internet Res. 2024 Dec 19;26:e57786. doi: 10.2196/57786 (PMC11695950; doi:10.2196/57786)
Supplement: Multimedia Appendix 2 [file jmir_v26i1e57786_app2.doc]

**Multimedia Appendix 2: Translated semi-structured interview guide.**

*Introduction of the interviewer and the research question*

*Introduction to the interview format*

**Part 1:** Demographic information (age, gender, profession, total work experience)

**Part 2:** Definition, processes, and requirements of a digitalized medical practice (as introductory questions)

- How would you define a digitalized medical practice? What do you associate with a digitalized medical practice?
- In your opinion, which processes in a medical practice are affected by digitalization? Follow-up question: Can you make a prioritization?
- What requirements must be met for a digitalized medical practice?

**Part 3: Dimensions of digital maturity in general practice**

- What specific factors/dimensions would you use to measure the level of digitalization of a medical practice? Under what conditions is one practice more digitally mature than another?
- What specific factors/dimensions relating to people in a medical practice would you use to assess the level of digitalization?
- What specific factors/dimensions relating to the organization of a medical practice would you use to measure the level of digitalization?
- What specific factors/dimensions relating to technologies and digital applications in a medical practice would you use to measure the level of digitalization?

*Something else?*

*Conclusion and goodbye*
