# Supplementary material for: Dimensions and Subcategories of Digital Maturity in General Practice: Qualitative Study
Source: J Med Internet Res. 2024 Dec 19;26:e57786. doi: 10.2196/57786 (PMC11695950; doi:10.2196/57786)
Supplement: Multimedia Appendix 3 [file jmir_v26i1e57786_app3.docx]

**Multimedia Appendix 3: Descriptions of dimensions and sub-categories of digital maturity in general practice**

| **Dimension** | **Description** | **Sub-category** | **Description** |
| --- | --- | --- | --- |
| **Deductively derived dimensions** | | | |
| **Digitally supported processes (1)** | Existence and extent of the use of digital solutions in medical practice processes | Patient processes | Core processes of the medical practice with patient contact, e. g. making appointments |
|  |  | Administrative and service processes | Internal administrative processes of the medical practice, e.g. purchasing, personnel, billing |
|  |  | Exchange with external cooperation partners | Digitalization of communication channels between the medical practice and external cooperation partners, e. g. other physicians |
| **Practice staff (2)** | Individual characteristics, skills, and abilities of the practice staff | Competences | Competencies of practice staff, e.g. correct use of digital systems |
|  |  | Willingness | Willingness of practice staff to use digital applications (correctly) |
|  |  | Mindset | Attitude of practice staff towards digital applications |
|  |  | Understanding of role | Integration of the use of digital systems in the professional understanding of practice staff |
| **Organizational structures and rules (3)** | Structures and rules in medical practices that are established at organizational level | Responsibilities | Existence and extent of responsibilities for digitalization projects |
|  |  | Corporate culture and policy | Anchoring the use of digital systems in the medical practice's mission statement |
|  |  | Participation | Extent of possible participation of practice staff in decisions on digitalization projects |
|  |  | Quality and knowledge management | Existence and extent of rules for the standardized use of digital systems including their electronic documentation |
|  |  | Change management | Handling and adaptability of the medical practice with changes in digitalization |
| **Technical infrastructure (4)** | Technical conditions and capabilities within the medical practice | Maturity of hardware and software | Performance, usability and stability of hardware and software |
|  |  | Interoperability | Possibility of communication and data exchange between different systems within the practice |
|  |  | Network infrastructure | Network speed, e.g. through broadband connection of the medical practice |
|  |  | IT security and data protection | Fulfillment of legal requirements for IT security and data protection. |
| **Inductively derived dimensions** | | | |
| **Benefit and outcome (5)** | Beneficial use of digitalization | Structures and processes | Beneficial use of digitalization associated with efficiency benefits and easier work for practice staff |
|  |  | Patient care | Beneficial use of digitalization associated with real added value for patients, e.g. through safer patient care |
|  |  | Finances | Beneficial use of digitalization associated with positive effects on key business figures |
| **External framework conditions (6)** | Influencing digitalization and its measurement through external framework conditions that cannot be influenced directly or with difficulty | Patient situation | The patient's situation, e.g. with regard to current care needs |
|  |  | Dependence on industry | Dependence on the industry, e.g. with regard to software and hardware |
|  |  | Maturity of external cooperation partners | Maturity level of the external cooperation partners, e.g. with regard to a possible electronic exchange |
|  |  | Financial framework conditions | Legal framework conditions in the country's healthcare system, e.g. on remuneration |
|  |  | Legal framework conditions | Framework conditions in the country's healthcare system, e.g. professional regulations |
|  |  | Practice size and form | Practice size and form of medical practice |
|  |  | Location | Location of medical practice |
